# Effects of early inertial exercises on morphological and functional recovery after ACL reconstruction

**DOI:** 10.1038/s41598-026-42441-y

**Published:** 2026-03-08

**Authors:** Alicja Naczk, Konrad Wieczorek, Paweł Kaczorowski, Piotr Gramza, Ewa Gajewska, Mariusz Naczk

**Affiliations:** 1 Department of Physical Education and Sport, Faculty of Physical Culture in Gorzow Wielkopolski, Poznan University of Physical Education, Poznan, Poland; 2 Rehakompleks Medical&Sport, Gorzow Wielkoposki, Poland; 3 Association of Lubusz Innovation Network, Gorzow Wielkoposki, Poland; 4 Department of Developmental Neurology, Poznan University of Medical Sciences, Poznan, Poland; 5 Department of Applied and Clinical Physiology, University of Zielona Gora, Collegium Medicum, Zielona Gora, Poland

**Keywords:** ACL reconstruction, Rehabilitation, Inertial training, Eccentric exercise, Anatomy, Health care, Medical research

## Abstract

Effective and safe rehabilitation following ACL reconstruction is an important factor in determining a patient’s return to health. This pilot randomized controlled trial aimed to compare the effectiveness of two rehabilitation protocols in patients following ACL reconstruction. Twenty-four adults following anterior cruciate ligament reconstruction were randomly allocated to the study groups. The first group (SR) underwent standard rehabilitation, whereas the experimental group (SRI) performed standard rehabilitation and additional inertial exercises starting from the seventh week. After 12 weeks of rehabilitation, isokinetic and inertial strength, dynamic balance, thigh circumference, and body composition were evaluated. At the post-intervention assessment, both groups demonstrated comparable isokinetic strength and dynamic balance performance, with no significant between-group differences. In contrast, the SRI group demonstrated greater restoration of morphological symmetry and interlimb strength symmetry under inertial loading compared with the SR group (*p* < 0.05; Cohen’s d = 1.37). Additionally, only the SRI group exhibited a significant increase in muscle mass of the operated limb, whereas no significant change was observed in the SR group. These findings indicate that early inclusion of inertial training provides additional benefits in morphological and inertial interlimb symmetry, without compromising conventional neuromuscular strength or balance outcomes following arthroscopic ACL reconstruction.

## Introduction

Anterior cruciate ligament (ACL) injury is one of the most common and limiting musculoskeletal conditions affecting active individuals, often resulting in long-term functional impairment^1,2^. Despite advances in surgical techniques and the implementation of standardized postoperative rehabilitation protocols, achieving full recovery of neuromuscular function and joint stability following ACL reconstruction surgery remains challenging. A return to pre-injury levels of functional independence and overall health is generally expected within 9–12 months^3^. However, persistent deficits in muscular strength of the operated limb have been reported even 24 months post-surgery^4^. Studies have shown that knee extensor strength can be 20–30% lower in the involved limb than in the uninvolved limb three months after surgery, and residual deficits of 10–15% are common after six months of rehabilitation^5,6^. Such asymmetries are clinically significant as they are associated with altered lower limb biomechanics, reduced functional capacity, and an increased risk of secondary injuries and post-traumatic osteoarthritis^7,8^. Insufficient strength, particularly in the quadriceps, has been linked to an increased risk of graft failure and long-term joint degeneration^6,7^. Reliable assessment of functional capacity after ACL reconstruction remains essential and has been recently validated in post-ACLR populations using standardized performance tests^9,10^.

Progressive resistance training, particularly focusing on the quadriceps, is widely accepted as an essential element of successful ACL rehabilitation^11,12^. Resistance training has been shown to improve muscle strength, restore neuromuscular function, reduce pain, and improve quality of life after ACL reconstruction^13,14^. Implementing strength training early within a structured protocol can enhance functional recovery and reduce asymmetries^2^. To return to performance and re-injury prevention after ACL reconstruction, there is a need to optimise rehabilitation processes^15,16^.

In recent years, inertial training (e.g. flywheel training) has gained attention due to its ability to induce eccentric overload and enhance force production^17–21^. Although the positive effects of inertial training on athletic^18^, healthy^20^, and elderly/disabled^22,23^ populations have been established, its application in post-ACLR rehabilitation remains relatively unexplored. Stojanović et al.^24^ demonstrated that eccentric-oriented flywheel training improved strength and functional performance in professional athletes; however, training was implemented in the late rehabilitation phase and in open kinetic chains. Patra et al.^25^ reported improvements in muscular endurance and eccentric power when isoinertial exercises were added to standard rehabilitation after ACL reconstruction. Most flywheel-based protocols are performed in open kinetic chain configurations, which may limit their applicability during early postoperative rehabilitation due to safety considerations. Contemporary consensus statements emphasize graft protection, criterion-based progression, and controlled strengthening during the early postoperative phase following ACL reconstruction^14–16^. Within this framework, structured rehabilitation commonly incorporates closed kinetic chain strengthening exercises. Therefore, inertial loading was implemented in a closed kinetic chain configuration to align with established early-stage rehabilitation principles. In the present study, “early inclusion” refers to the initiation of inertial exercises at the beginning of week 7, corresponding to the period immediately following the commonly described early protective phase (approximately 0–6 weeks) in ACL rehabilitation literature.

This study aimed to evaluate the effects of including inertial knee extension training using a novel device (InerKnee) in a standard rehabilitation program on muscle strength, thigh morphology, body composition, and dynamic balance following anterior cruciate ligament reconstruction. We hypothesized that adding inertial training would improve rehabilitation outcomes, particularly quadriceps recovery and interlimb symmetry, compared with standard rehabilitation alone.

## Methods

### Participants

A group of 24 adults (5 women and 19 men; age, 36.7 ± 11.1 years, range 18–56 years; body mass, 86.7 ± 20.9 kg; height, 175.2 ± 10.2 cm) after ACL reconstruction met the inclusion criteria and were included in the study. Inclusion criteria: adult men and women who had sustained an acute knee injury resulting in a complete anterior cruciate ligament (ACL) rupture, confirmed by magnetic resonance imaging (MRI) and clinical examination. Eligible participants underwent ACL reconstruction within a period ranging from the day of injury up to 12 months post-injury. All participants were medically cleared to begin a structured postoperative rehabilitation program. Exclusion criteria - participants were excluded if they presented with any of the following conditions: history of previous knee surgery or ACL reconstruction on the same (injured) limb, concomitant injury to other major knee structures (e.g., posterior cruciate ligament, collateral ligaments, menisci requiring repair, articular cartilage damage), chronic or degenerative joint diseases (e.g., osteoarthritis, rheumatoid arthritis), neuromuscular or musculoskeletal disorders affecting lower limb function, systemic diseases influencing muscle or connective tissue function, any contraindications to physical exercise or participation in resistance training, or lack of adherence to the full rehabilitation protocol. In addition, patients requiring multistructural or multitissue surgical procedures were excluded. Multiligamentous knee injuries, articular cartilage damage, or unstable meniscal tears constituted exclusion criteria. The presence of a concomitant stable medial meniscal tear was not considered an exclusion criterion, provided that it did not require surgical repair or modification of postoperative recommendations and the standardized rehabilitation protocol.

**Fig. 1:**
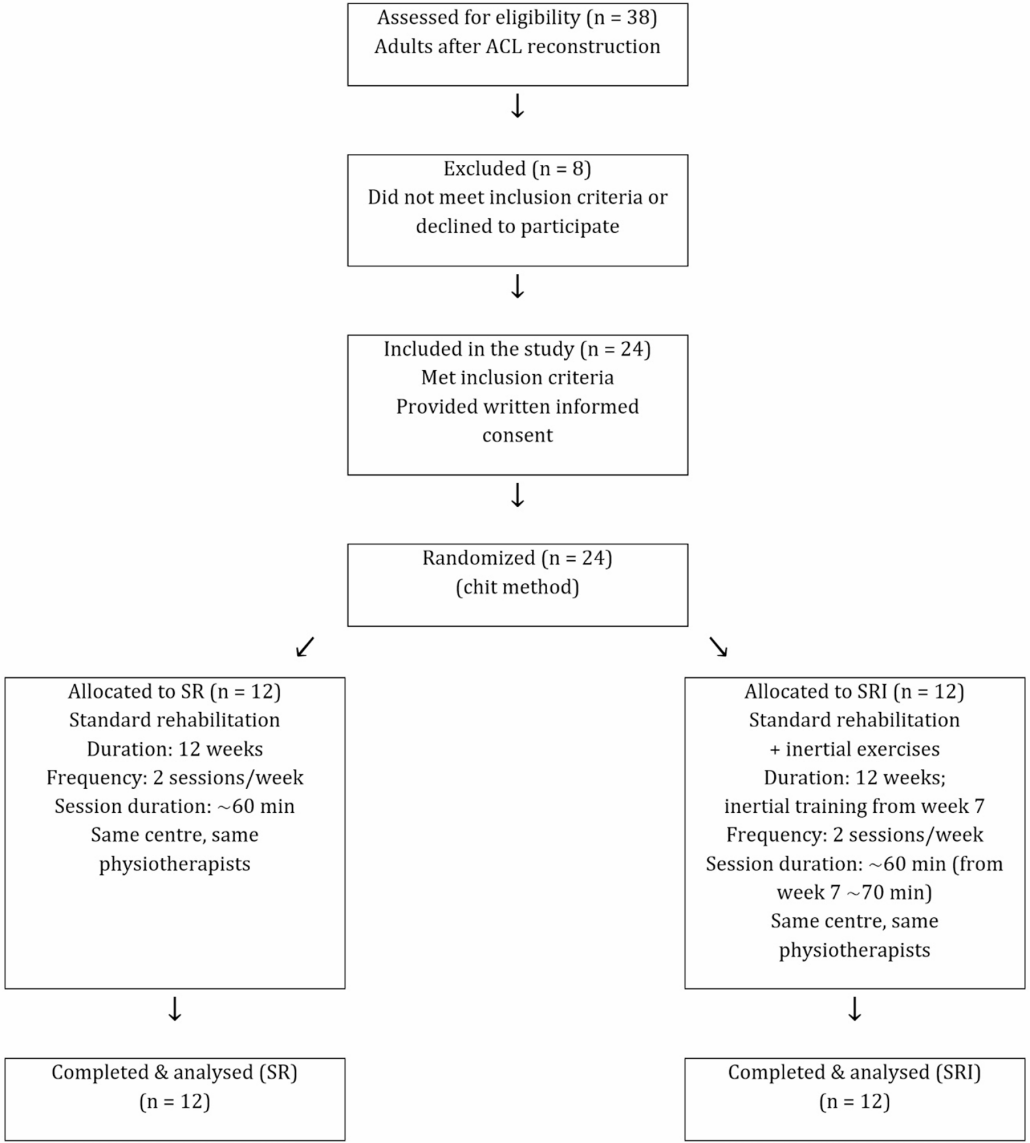
Flow diagram of participants.

The participants were randomly allocated into two groups: a standard rehabilitation group (SR; *n* = 12) and a standard rehabilitation plus inertial exercises group (SRI; *n* = 12). (Table 1). Participants were randomly allocated to the SR or SRI group using simple randomization (lottery method). After eligibility was confirmed (24 of 38 screened participants), group assignment was performed by an independent person not involved in recruitment, intervention delivery, or outcome assessment. Randomization was conducted prior to baseline assessments. Due to the nature of the intervention, participants and physiotherapists were not blinded; however, outcome assessments were conducted by two independent assessors blinded to group allocation. Each participant was qualified for ACL reconstruction surgery as a result of different acute injuries (football: *n* = 12, skiing: *n* = 4, basketball: *n* = 1, volleyball: *n* = 1, kickboxing: *n* = 2, traffic accident: *n* = 3, and playing with children: *n* = 1). Participant recruitment, group allocation, follow-up, and analysis are summarized in a flow diagram, (Fig. 1). All surgeries were performed by the same physician using the same procedure (described below). Each subject participated in 12-week rehabilitation in the same rehabilitation company. Rehabilitation was carried out by the same two physiotherapists twice a week for approximately 60 min per session. Subjects from the SR group performed standard rehabilitation applied by the rehabilitation company (described below), while SRI performed the same rehabilitation and additionally performed inertial exercises from the seventh week of rehabilitation. This study was designed as a pilot randomized controlled trial. Given its exploratory nature, no formal a priori sample size calculations were performed. The sample size was determined pragmatically based on the number of eligible patients who met the inclusion criteria and were recruited during the predefined study period. This study was approved by the Bioethics Committee of the Collegium Medicum University of Zielona Góra (approval no. RCM-CM-KBUZ031.13.2022), Poland. All procedures were performed in accordance with the Declaration of Helsinki and relevant national regulations. Written informed consent was obtained from all participants prior to participation. The trial was prospectively registered at ClinicalTrials.gov (identifier: NCT06726044; date of registration: 12.05.2024).

**Table 1:** Baseline characteristics of subjects.

Variable	SRI (*n* = 12)	SR (*n* = 12)	*p*-value	Cohen’s d
Age (years)	36.9 ± 10.3	36.8 ± 10.3	> 0.9	< 0.01
Height (cm)	175.4 ± 10.4	175.4 ± 10.4	> 0.9	< 0.01
Body mass (kg)	89.5 ± 25.6	88.3 ± 24.7	> 0.8	0.05
BMI (kg/m²)	28.6 ± 5.5	28.2 ± 5.3	> 0.7	0.07

Values are mean ± SD, p-value and Cohen’s d - between groups.

### Surgical technique

All anterior cruciate ligament reconstructions were performed arthroscopically with the patient in the supine position under tourniquet control. An autograft harvested from the semitendinosus and gracilis tendons was used in each case. After harvesting, the grafts were appropriately prepared and configured for implantation into femoral and tibial bone tunnels. No synthetic tape or suture-augmentation devices were used. Following standard arthroscopic inspection of the knee joint, femoral and tibial tunnels were created using dedicated instruments. Femoral fixation was achieved using a cortical suspension device (Ultrabutton, Smith & Nephew), and tibial fixation was performed using a titanium interference screw (SoftSilk, Smith & Nephew). In cases where a concomitant stable medial meniscal tear was identified, meniscal repair was performed using an all-inside technique with the Fast-Fix 360 system (Smith & Nephew). All patients received a compressive dressing and a surgical drain, which was removed on the first postoperative day. The skin sutures were removed 14 days after the surgery.

### Rehabilitation

All participants completed a 12-week standard rehabilitation program including manual therapy (mobilization of the patella and fibular head, myofascial release, manual scar treatment, osteopathic techniques), anti-swelling interventions (manual lymphatic drainage, compression, cryotherapy), kinesiotaping, joint activation exercises, flossing, and kinesiotherapy. Exercises included strength training and knee flexion–extension performed in both closed and open kinetic chains, as well as isometric quadriceps exercises through the full range of motion.

**Fig. 2:**
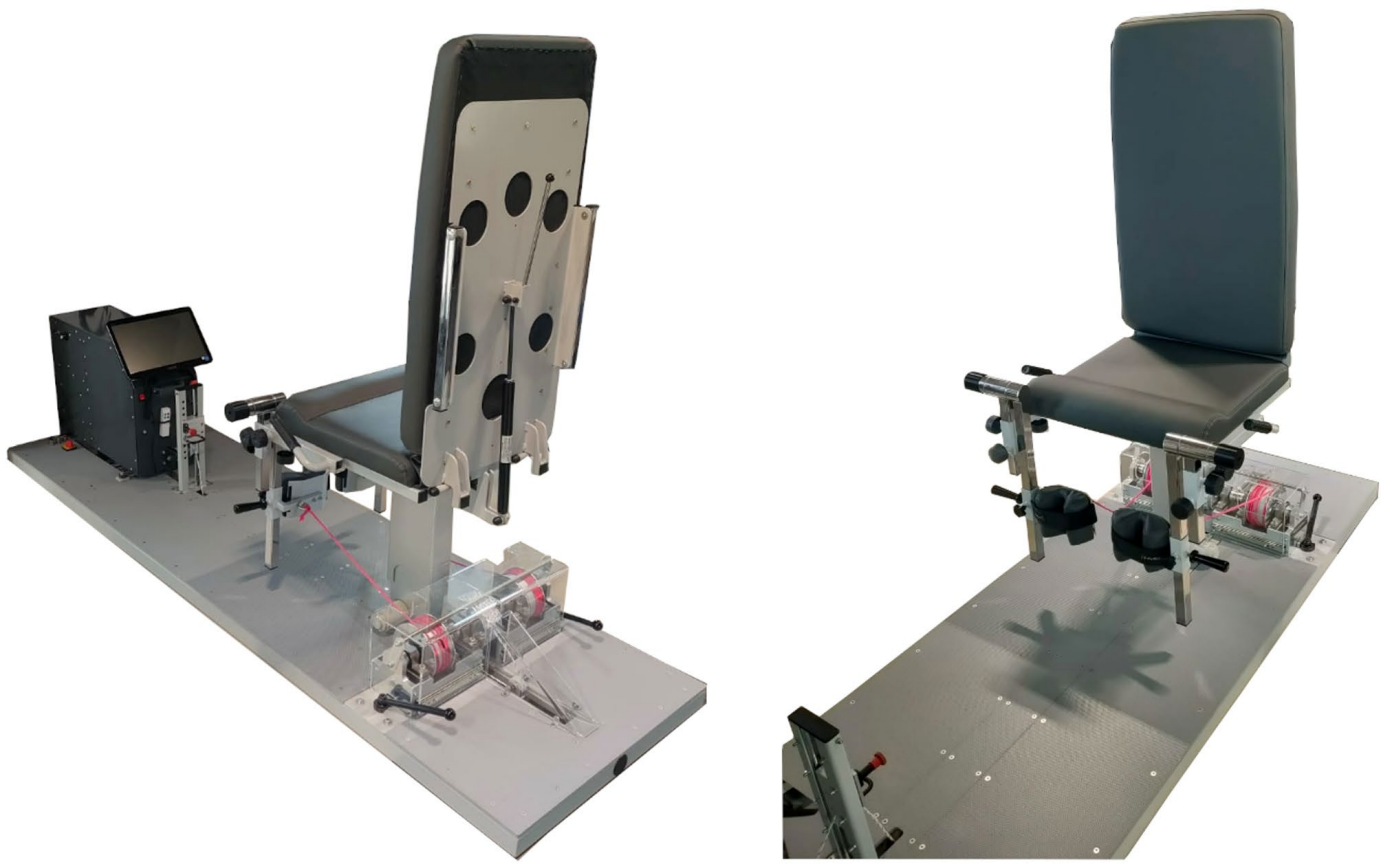
InerKnee - inertial rehabilitation device.

From the seventh to the twelfth week of rehabilitation subjects from SRI additionally to standard rehabilitation program performed inertial exercises. Due to safety considerations during the early postoperative phase, inertial exercises were deliberately implemented in a closed kinetic chain configuration to ensure controlled load transmission across the knee joint. For this purpose, a dedicated inertial rehabilitation device (InerKnee - Fig. 2) was designed and manufactured within the framework of the present research project to enable inertial knee extension training under closed kinetic chain conditions in a seated position. More details concerning this device are provided in Strength measurements section. Inertial exercises were performed twice a week, 4 sets of exercises, each lasting 15 s, 2 min rest periods between sets were used. Range of motion was 60 degrees. Before including inertial exercises to rehabilitation maximal strength of knee extensors of uninvolved limb under inertial conditions was evaluated. During the first two weeks of inertial exercises performed by involved limb a load of 60% of the maximal strength obtained by the uninvolved limb was used. Then, every two weeks of inertial exercises the load was increased by 10% so during the last two weeks it was 80% of the maximum force of the uninvolved limb. All inertial exercises were performed under the same physiotherapist supervision to ensure proper technique and safety.

### Measurements

Baseline assessments were performed two weeks post-surgery and included body height, body mass, thigh circumference, and body composition. Pre-intervention strength testing of the operated limb was not performed due to safety considerations in the early postoperative phase, when maximal voluntary efforts could potentially compromise graft protection and patient comfort. After rehabilitation, these measures were repeated, and additional assessments of isokinetic knee extensor and flexor strengths, inertial knee extensor strength, and dynamic balance were performed.

### Thigh circumference

Measurement of thigh circumference was accomplished with the patient in the supine position with the knee fully extended. Both the uninvolved and the involved limb were assessed. The circumference of the thigh was measured with a tape measure at points of 5.0 cm, 10.0 cm, and 15.0 cm, proximal to the superior pole of the patella, by the same examiner with each made to the nearest 0.5 cm. According to the protocol used by Yoshii et al.^26^, the thigh circumference was assessed at 5, 10, and 15 cm above the patellar border. However, during measurements, 5 cm above the patella, a swelling effect was observed, resulting in disproportionately high early postoperative circumferences that did not represent true muscular changes following rehabilitation. Therefore, only the 10- and 15-cm measurements were considered and included in the primary statistical analysis. The same researcher took three measurements, with each made to the nearest 0.5 cm. The mean value of the three measurements was used for future calculations.

### Body composition

To evaluate the influence of rehabilitation on body composition, a bioelectrical impedance device (Tanita MC-980 MA; Tanita Corporation, Tokyo, Japan) was used. The subjects were asked to maintain a normal state of hydration prior to the measurements, and they were not allowed to exercise or eat for 12 h preceding the measurements. The measurements were made in the morning according to the manufacturer’s guidelines.

### Strength measurements

Maximal voluntary torque (MVT) under isokinetic conditions.

The maximal torque derived from isokinetic muscle actions was determined using a specialized Biodex 4 Pro device (Shirley, NY, USA). Data collection was preceded by a familiarization session. Measurements were collected in a seated position. During measurements, the ankle of the active leg was attached to the Biodex 4 Pro device moving shin pad. In the starting position, the thigh of the active leg was immobilized at 90 degrees in relation to the trunk, and the knee was positioned at 60 degrees (180 degrees corresponded to complete maximal extension of the knee joint). To prevent any activity of other muscle groups that were not being tested, the participant’s trunk was stabilized using belts across the chest. Measurements were performed at two velocities 60°/s and 180°/s with a 2-min rest period between measurements. During both measurements, first the healthy leg and then the operated leg were tested. Each measurement began with two trial cycles, each composed of extension and flexion of the knee joint, followed by five repetitions involving maximal strength. Prior to the measurements, the participants were given verbal instructions regarding the experiment’s design. The highest value among the five maximal trials of each measurement was adopted for further analysis.

The maximal force under inertial conditions.

The measurements were made using InerKnee device (Fig. 2). This device was designed and manufactured as part of a publicly funded research and development project focused on the development of an innovative, inertial rehabilitation protocol following arthroscopic ACL reconstruction. The device concept was informed by prior inertial systems. Unlike common flywheel systems typically used in open kinetic chain configurations, InerKnee enables inertial loading under closed kinetic chain conditions. The device demonstrates high test–retest reliability (intraclass correlation coefficient consistency ≥ 0.941, ICC agreement ≥ 0.923). Measurements were preceded by two familiarization sessions to ensure proper technique and minimize potential task-specific learning effects. After warm-up, each participant performed a 10-second maximal test of the knee extensors. First test was performed at the start of inertial rehabilitation (7th week of rehabilitation) and only healthy leg was tested. At the end of rehabilitation first the healthy leg and then the operated leg were tested with a 1-minute break between measurements. All tests were performed while seated in a rehabilitation chair. The range of motion for each exercise was approximately 60 degrees. All measured parameters (force, range of motion, etc.) were recorded using a computer and displayed in real time on the screen. The maximal value of force (in newtons) achieved during one full cycle was used for further analysis.

### Star Excursion Balance Test (SEBT)

Dynamic balance was assessed using the SEBT according to the methodology described by Bulow et al.^27^. Participants completed a warm-up consisting of five minutes of brisk walking on a treadmill, followed by a dynamic stretching routine. The patients performed three practice trials and three measurement trials of the SEBT for both legs. The average of the three measurements was used for the data analysis (in cm). The patients stood barefoot with both hands placed on their hips. The patients were instructed to reach their foot and touch the tape as far as possible in the anterior (ANT), posteromedial (PM), and posterolateral (PL). A standardized order of testing was utilized, the uninvolved limb was measured first in the order of ANT, PM and PL. Testing was repeated in the same order for the involved stance limb. The tester provided patient feedback and proper guidance to reduce mistakes during practice trials. The SEBT - ANT, PM, PL, and composite scores were used for the analyses. The SEBT scores were normalized to the limb length measurement of the patients.

All interventions were well tolerated, and no adverse events were observed during the rehabilitation period.

### Statistical analysis

The Shapiro–Wilk test was used to assess the normality of data distribution. Descriptive statistics are presented as means ± standard deviations. For variables assessed both before and after the intervention (thigh circumference and body composition parameters), a two-way repeated-measures analysis of variance (RM-ANOVA) was performed, with time (pre vs. post) as the within-subject factor and group (SR vs. SRI) as the between-subject factor. When significant main effects or interactions were detected, the Scheffé post hoc test was used to identify pairwise differences. To further characterize the magnitude of training effects, relative changes (%) were calculated for each participant. Between-group differences in relative changes were analyzed using independent-samples t-tests. For outcomes assessed only after rehabilitation (isokinetic strength, inertial strength, and dynamic balance), between-group comparisons were conducted using independent-samples t-tests.

Effect sizes (ES) were calculated using Cohen’s d. Within-group effect sizes were computed for pre–post comparisons using dependent-samples Cohen’s d, whereas between-group effect sizes were calculated based on relative change (%) scores. Effect sizes were interpreted according to Cohen’s criteria: d < 0.41 (small), 0.41–0.70 (moderate), and > 0.70 (large)^28^. The level of statistical significance was set at *p* ≤ 0.05.

## Results

### Thigh circumference

As described in the Methods, thigh circumference analyses were limited to measurements obtained at 10 and 15 cm above the patella (Table 2). At baseline, both groups demonstrated significant interlimb asymmetry at both measurement levels. After rehabilitation, differences between the SR and SRI groups were observed. In the SRI group, interlimb asymmetry was substantially reduced at both 10 and 15 cm, and post-intervention differences between the operated and healthy limbs were no longer statistically significant, accompanied by moderate-to-large effect sizes. In contrast, the SR group demonstrated only limited reductions in asymmetry, with statistically significant interlimb differences persisting after rehabilitation and small effect sizes. Overall, morphological symmetry was restored only in the SRI group.

**Table 2:** Thigh circumference 10 and 15 cm above the patella before and after rehabilitation.

Distance above patella	Group	OP pre (cm)	NOP pre (cm)	Asymmetry pre (cm)	OP post (cm)	NOP post (cm)	Asymmetry post (cm)	Cohen’s d (within)	Δ Asymmetry (cm)	Cohen’s d (between)	*p* (between-group Δ)
10 cm	SRI	48.9 ± 6.4	50.8 ± 6.4	1.92 ± 1.58*	51.0 ± 7.0	51.6 ± 6.3	0.67 ± 2.25	0.64	− 1.25	0.72	0.02
	SR	48.0 ± 5.2	49.6 ± 4.5	1.67 ± 1.60*	49.3 ± 4.5	50.7 ± 3.9	1.46 ± 1.30#	0.14	− 0.21	–	–
15 cm	SRI	53.7 ± 6.9	55.3 ± 6.3	1.61 ± 1.21*	56.3 ± 7.2	56.8 ± 6.6	0.50 ± 1.31	0.88	− 1.11	0.68	0.03
	SR	52.4 ± 5.6	54.6 ± 4.9	2.21 ± 1.40*	53.8 ± 4.5	55.5 ± 4.1	1.63 ± 1.30#	0.43	− 0.58	–	–

Interlimb asymmetry calculated as non-operated minus operated thigh. Measurements at 5 cm were excluded from the primary analysis because of postoperative swelling, OP- operated limb, NOP – non-operated limb, * significant difference between operated and non-operated thigh within group before rehabilitation, # significant difference between operated and non-operated thigh within group after rehabilitation.

### Muscle strength

#### Isokinetic conditions

Isokinetic knee extensor and flexor strength assessed at 60°/s and 180°/s revealed persistent strength deficits in the operated limb relative to the healthy limb in both groups after rehabilitation (Table 3). The magnitude of extensor deficits was greater than that of flexor deficits across velocities. Between-group comparisons showed no statistically significant differences in strength asymmetry at either angular velocity, with small effect sizes, indicating comparable post-rehabilitation isokinetic strength outcomes in the SR and SRI groups.

**Table 3:** Isokinetic strength deficits after rehabilitation (Biodex).

Velocity	Muscle group	SRI OP (Nm)	SRI NOP (Nm)	SRI Deficit (%)	SR OP (Nm)	SR NOP (Nm)	SR Deficit (%)	*p* (SRI vs. SR)	Cohen’s d
60°/s	Extensors	161.1 ± 39.4	215.2 ± 44.5	− 25.5 ± 6.8*	171.8 ± 59.9	231.0 ± 64.9	− 26.9 ± 12.7*	0.88	0.11
	Flexors	89.6 ± 19.1	104.4 ± 20.5	− 13.8 ± 10.5	86.7 ± 27.9	104.9 ± 31.7	− 16.8 ± 19.1	0.74	0.16
180°/s	Extensors	114.4 ± 32.3	140.9 ± 39.0	− 18.3 ± 9.3	121.8 ± 42.0	141.7 ± 39.9	− 15.5 ± 16.1	0.69	0.18
	Flexors	70.7 ± 21.6	79.3 ± 20.4	− 10.8 ± 11.5	68.5 ± 21.4	75.4 ± 21.5	− 8.3 ± 20.6	0.81	0.09

*Significant difference between operated and non-operated thigh within group after rehabilitation.

### Inertial conditions

Under inertial conditions, interlimb strength asymmetry was observed in both groups (Table 4). However, between-group analysis showed significantly greater operated-to-healthy limb symmetry in the SRI group compared with the SR group. This difference was associated with a large effect size, indicating superior restoration of strength symmetry following rehabilitation that included inertial exercises.

**Table 4:** Inertial strength deficits after rehabilitation (InerKnee).

Group	OP (*N*)	NOP (*N*)	LSI	Deficit (%)	Cohen’s d	*p*
SRI	263 ± 40.8	286 ± 41.0	0.92 ± 0.03	8.0 ± 3.1	1.37	0.004
SR	257 ± 64.0	293 ± 71.5	0.88 ± 0.04	12.0 ± 4.5		

LSI – limb symmetry index operated limb/non-operated limb.

### Dynamic Balance (SEBT)

Dynamic balance assessed using the SEBT showed no statistically significant interlimb differences in either group across all reach directions (Table 5). Additionally, no between-group differences were observed in percentage interlimb asymmetry, indicating comparable restoration of dynamic postural control in both rehabilitation protocols.

**Table 5:** Dynamic balance (SEBT) after rehabilitation.

Direction	Group	Operated limb (cm)	Healthy limb (cm)	LSI (%)	*p* (within)	*p* (between)
ANT	SRI	70.0 ± 8.0	69.0 ± 8.5	101.4	0.74	0.98
	SR	75.0 ± 9.7	74.0 ± 8.5	101.3	0.70	
PM	SRI	99.0 ± 9.3	101.0 ± 11.3	98.0	0.66	0.75
	SR	105.0 ± 10.5	106.0 ± 8.8	99.1	0.89	
PL	SRI	98.0 ± 10.1	100.0 ± 10.8	98.0	0.61	0.18
	SR	101.0 ± 15.2	101.0 ± 13.6	100.0	0.88	

### Body composition

At baseline, no significant between-group differences were observed for any body composition variable (Table 6). Over the 12-week rehabilitation period, the SRI group demonstrated significant reductions in body mass and adiposity-related parameters, along with a significant increase in muscle mass of the rehabilitated limb. No significant changes were observed in the non-operated limb. In contrast, the SR group showed no significant pre–post changes in body mass, adiposity, fat-free mass, or segmental muscle mass in either limb.

**Table 6:** Body composition before and after rehabilitation.

Parameter	SRI Pre	SRI Post	Δ SRI	*p* (SRI)	SR Pre	SR Post	Δ SR	*p* (SR)
Body mass (kg)	89.52 ± 25.55	88.34 ± 24.67	− 1.18	0.03	86.40 ± 18.28	85.85 ± 18.63	− 0.55	0.52
BMI (kg/m²)	28.63 ± 5.48	28.23 ± 5.30	− 0.39	0.04	28.35 ± 5.58	28.13 ± 5.54	− 0.22	0.42
Fat mass (%)	26.59 ± 6.28	24.93 ± 6.96	− 1.66	0.01	23.31 ± 9.26	23.22 ± 10.01	− 0.09	0.84
Fat mass (kg)	24.36 ± 11.46	22.46 ± 10.56	− 1.90	0.01	20.91 ± 10.94	20.62 ± 11.38	− 0.29	0.60
Visceral fat level	8.83 ± 5.37	7.75 ± 5.33	− 1.08	0.03	7.75 ± 5.77	7.92 ± 6.11	+ 0.17	0.50
Fat-free mass (kg)	65.08 ± 16.22	65.88 ± 16.76	+ 0.80	0.11	65.49 ± 11.71	65.13 ± 11.87	− 0.36	0.47
Muscle mass – rehabilitated limb (kg)	10.73 ± 3.10	11.26 ± 3.49	+ 0.53	0.03	10.80 ± 2.10	10.99 ± 2.24	+ 0.19	0.19
Muscle mass – healthy limb (kg)	10.70 ± 3.10	10.63 ± 3.10	− 0.07	0.44	10.75 ± 2.26	10.72 ± 2.33	− 0.03	0.79

Values are presented as mean ± SD; *p* values refer to within-group pre–post comparisons.

## Discussion

The present findings partially support the initial hypothesis, indicating that the inclusion of inertial exercises in the early rehabilitation phase was associated with greater improvements in morphological symmetry and interlimb strength symmetry assessed under inertial loading compared with standard rehabilitation, while isokinetic strength and dynamic balance outcomes were comparable between groups at 12 weeks.

Although both interventions contributed to a reduction in thigh circumference asymmetry, near-normalization of interlimb differences was observed exclusively in the SRI group. In addition, a significant increase in muscle mass was detected only in the operated limb of the SRI group, whereas no significant changes were observed in the SR group. Persistent asymmetry in muscle strength and size after anterior cruciate ligament (ACL) reconstruction has been associated with altered movement mechanics, abnormal landing biomechanics, and worse functional outcomes, underscoring the clinical relevance of symmetrical quadriceps recovery^29,30^. Reduced quadriceps muscle thickness and cross-sectional area have also been reported to persist for months after surgery and to correlate with delayed functional recovery^31,32^.

Therefore, our findings indicate that the inclusion of inertial exercises in rehabilitation after ACL reconstruction was associated with greater restoration of interlimb symmetry and improvements in quadriceps muscle morphology. A distinctive feature of our study was the use of a purpose-built device enabling inertial knee extension training under closed kinetic chain conditions. This device was created for safety during early rehabilitation and may have facilitated the application of eccentric-oriented inertial loading without adversely affecting conventional strength or balance outcomes.

The superiority of inertial training may be explained by the mechanical characteristics of flywheel-based exercise, which provides high eccentric loading proportional to concentric effort. Eccentric overload is a key stimulus for muscle hypertrophy and neural adaptations, particularly in the presence of arthrogenic muscle inhibition after ACL reconstruction^33,34^. Conventional resistance training may not provide comparable eccentric stimuli, potentially limiting morphological recovery^6,35^.

In both groups, isokinetic testing revealed persistent strength deficits in the operated limb compared with the healthy limb, with greater deficits in knee extensors than flexors. At 12 weeks, isokinetic strength asymmetry was similar between groups, suggesting that the six-week inertial phase was not reflected in peak torque measures at this early postoperative stage. Because isokinetic testing of the operated limb before rehabilitation was not feasible for safety reasons, the present analysis reflects end-state neuromuscular capacity rather than recovery trajectories.

Despite comparable post-rehabilitation strength asymmetry, the SRI protocol may have influenced recovery through mechanisms that were not fully captured by isokinetic peak torque measurements. Isokinetic dynamometry primarily quantifies maximal voluntary torque under controlled angular velocities, whereas inertial resistance training emphasizes eccentric overload, rapid force absorption, and neuromuscular coordination under variable loading conditions. Such adaptations may preferentially affect functional performance, rate of force development, and intermuscular coordination, rather than maximal concentric torque alone.

From a clinical perspective, the persistence of strength deficits of approximately 15–30% after 12 weeks of rehabilitation is consistent with previous reports following anterior cruciate ligament reconstruction and highlights the challenge of achieving full bilateral symmetry within standard early rehabilitation timelines. Importantly, the absence of exacerbated deficits in the SRI group indicates that the inclusion of inertial exercises is safe and does not compromise strength recovery, while potentially offering complementary benefits at the morphological level, as suggested by the greater reduction in thigh circumference asymmetry and greater increase in muscle mass in the operated limb observed in this group.

The strength deficits observed in both groups appear comparable to, or in some cases more favorable than, those reported at similar postoperative time points. Czaplicki et al.^36^ reported larger quadriceps deficits at approximately three months post-surgery, and Kim et al.^37^ observed persistent quadriceps deficits following a 12-week postoperative program. In comparison, the deficits observed in the present study fell within or below these ranges, despite the absence of a formal prehabilitation phase.

Taken together, these findings suggest that isokinetic peak torque alone may be insufficient to fully characterize the effects of inertial resistance training during the early postoperative phase of rehabilitation. At approximately three months post-surgery, strength recovery remains constrained by neuromuscular inhibition, incomplete morphological restoration, and conservative loading strategies; under such conditions, modality-specific advantages of inertial training may become more apparent with longer interventions, higher training doses, or during later and higher-intensity rehabilitation phases.

A novel aspect of this study is the assessment of knee extensor strength under inertial conditions using a closed kinetic chain configuration. To our knowledge, this is the first study to evaluate interlimb strength asymmetry under these conditions after ACL reconstruction. Strength asymmetry assessed under inertial loading was smaller than that observed during isokinetic testing, suggesting that isokinetic measures may overestimate functional asymmetry in early rehabilitation phases. Notably, interlimb symmetry under inertial conditions was significantly greater in the SRI group than in the SR group, despite similar absolute strength values. This likely reflects the neuromechanical demands of inertial loading, which impose high eccentric demands and continuous force modulation^38,39^. Improved symmetry under inertial conditions may better prepare patients for deceleration-based tasks encountered in later rehabilitation stages, supporting the inclusion of inertial resistance training as a complementary strategy following ACL reconstruction. Although formal minimal clinically important difference (MCID) values have not been established for inertial strength symmetry or segmental muscle mass following ACL reconstruction, the restoration of interlimb symmetry is widely recognized as clinically relevant in ACL rehabilitation. In particular, interlimb asymmetry below 10% is commonly used as a functional benchmark for return-to-sport decision-making. Therefore, the greater restoration of symmetry observed in the SRI group, accompanied by a large effect size (Cohen’s d = 1.37), may indicate a clinically meaningful improvement beyond statistical significance.

No significant interlimb asymmetry or between-group differences were observed in dynamic balance assessed by the SEBT after 12 weeks of rehabilitation. These findings suggest that both rehabilitation approaches were equally effective in restoring dynamic postural control at this stage of recovery. The normalization of SEBT performance is clinically relevant, as balance deficits are commonly reported early after ACL reconstruction and are associated with impaired neuromuscular control. These findings are consistent with previous reports, including those of Patra et al.^25^.

The results of this study may be applicable to adult patients undergoing primary arthroscopic ACL reconstruction who participate in a structured postoperative rehabilitation program supervised by qualified physiotherapists in routine clinical practice.

Several limitations should be acknowledged. The relatively small sample size may have limited statistical power to detect subtle between-group differences, particularly for outcomes with high interindividual variability. This limited statistical power increases the risk of Type II error. The follow-up period was restricted to the early postoperative phase, and strength, morphology, and neuromuscular control continue to evolve beyond 12 weeks. As the follow-up was limited to 12 weeks, the findings reflect early-stage rehabilitation outcomes and should not be extrapolated to later-stage functional recovery. Pre-rehabilitation assessment of the operated limb was not feasible for safety reasons, limiting analyses to post-rehabilitation comparisons. In addition, inertial exercises and strength testing were performed using the same device, and a task-specific learning effect cannot be fully excluded. Finally, morphological outcomes were assessed using segmental bioelectrical impedance analysis, which provides indirect estimates of muscle mass. Imaging-based techniques such as MRI or ultrasound would allow more precise evaluation of muscle morphology.

## Conclusions

Early inclusion of inertial training into standard rehabilitation after ACL reconstruction provides additional benefits in restoring morphological symmetry and interlimb strength symmetry assessed under inertial loading. Importantly, these adaptations were achieved without compromising conventional neuromuscular strength or dynamic balance outcomes, supporting the use of inertial training performed in closed kinetic chain as a complementary strategy during the early postoperative rehabilitation phase.

## Data Availability

The data analysed during the current study are publicly available in an open data repository at [https://doi.org/10.18150/XZNGVP].
